# Persistent bothersome urinary frequency following stereotactic body radiation therapy for clinically localized prostate cancer: rationale for prophylactic β3-agonist in men with elevated baseline international prostate symptom scores

**DOI:** 10.3389/fonc.2025.1642614

**Published:** 2026-01-15

**Authors:** Jennifer Zack, Min Ji Koh, Emily Lindbloom, Timothy O’Connor, Kelly Gaudian, Alan Zwart, Malika Danner, Deepak Kumar, Simeng Suy, Ryan Hankins, Sean Collins

**Affiliations:** 1 School of Medicine, Georgetown University, Washington, DC, United States; 2 Department of Radiation Oncology, Georgetown University Hospital, Washington, DC, United States; 3 Biotechnology Research Institute, North Carolina Central University, Durham, NC, United States; 4 Department of Radiation Oncology, University of South Florida (USF) Health Morsani College of Medicine, Tampa, FL, United States; 5 Department of Urology, Georgetown University Hospital, Washington, DC, United States

**Keywords:** prostate, prostate cancer, IPSS, LUTS (lower urinary tract symptoms), genitourinary toxicity (GU), beta 3 adreno-receptor agonists, SBRT (stereotactic body radiation therapy)

## Abstract

**Introduction:**

Stereotactic body radiation therapy (SBRT) for clinically localized prostate cancer has been associated with prolonged acute obstructive and irritative lower urinary tract symptoms (LUTS). Prophylactic alpha-adrenergic antagonists have been used therapeutically to prevent obstructive symptoms in patients undergoing prostate SBRT, however irritative symptoms may be better addressed with beta 3-adrenergic receptor agonists, which are shown to be safe and effective in men with overactive bladder (OAB).

**Objective:**

This study retrospectively examines the pattern of bothersome urinary frequency associated with SBRT to determine which patients would have potentially benefitted from prophylactic beta 3-adrenergic receptor agonist.

**Methods:**

Patients with clinically localized prostate cancer who underwent prostate SBRT (n=1676) were followed for 3 months post-treatment to evaluate for bothersome urinary frequency, which was assessed by question 4E on the EPIC-26. Answers to question 4E and tamsulosin usage were recorded at regular follow-ups to assess, and demographic factors as well as baseline prostate volume, alpha-adrenergic antagonist usage and IPSS score severity were used as modifiers.

**Results:**

Using the IPSS questionnaire to determine baseline LUTS, most patients reported moderate urinary symptoms (53%), followed by mild (34%) and severe (13%). Patients endorsed increased irritative LUTS at 1-month post-SBRT, with similar rates of frequency at 3 months compared to baseline. Patients with moderate and severe urinary symptoms per the baseline IPSS were more likely to endorse bothersome urinary frequency at one month post SBRT (EPIC-26 Q4E; OR 2.58 and 10.2 respectively) compared to those with mild symptoms. No significant differences were found in urinary frequency between patients who used and did not use an alpha antagonist at baseline.

**Conclusions:**

Bothersome post-SBRT urinary frequency persists 1-month post-SBRT. Baseline LUTS predicts bothersome post-treatment urinary frequency. Prophylactic alpha-adrenergic antagonists do not protect against bothersome acute urinary frequency. This paper makes the case that urinary frequency may be better addressed with a prophylactic beta-3 agonist, like vibegron, which is designed to treat storage-related LUTS concerns.

## Introduction

Stereotactic body radiation therapy (SBRT) is an increasingly popular intervention for the treatment of clinically localized prostate cancer (1–3). As more patients are choosing this convenient mode of treatment, it becomes imperative that potential side effects are well understood in order to guide symptom management. Lower urinary tract symptoms (LUTS) is a general term that includes the categories of obstructive (e.g., retention and hesitancy) and irritative (e.g., frequency, urgency, and urge incontinence) (4). Previous research has demonstrated an increased prevalence of acute LUTS in men undergoing prostate SBRT for up to 3 months post-treatment (5, 6). Prophylactic alpha-adrenergic antagonists are typically used as standard of care to treat obstructive symptoms, either prophylactically or at the earliest sign of symptoms (6–8). Irritative symptoms, however, may be better addressed by alternative strategies (5).

Irritative voiding symptoms are a common problem of aging (9). In men > 70 years old, the prevalence is greater than 20% (10). An enlarged prostate may increase the risk of bothersome irritative voiding symptoms (6, 11). Anti-cholinergic medications may relieve symptoms but are commonly discontinued due to bothersome dry mouth and/or constipation (12, 13). Beta-3 agonists are a newer effective medication class for overactive bladder (OAB) with an improved side effect profile and adherence (14). Beta-3 agonists relax bladder smooth muscle which increases storage capacity and prolongs intervals between voiding (15). In men with BPH and OAB, treatment beta-3 agonists did not adversely affect voiding parameters (16, 17).

The aim of this study was to retrospectively examine the pattern of bothersome urinary frequency in patients with clinically localized prostate cancer undergoing SBRT from the first day of treatment up to 3 months post-treatment. Additionally, we wished to evaluate the impact of baseline patient characteristics on post-SBRT bothersome urinary frequency, specifically at 1-month post-SBRT when we expect urinary frequency to be the most pronounced.

## Methods

### Patient selection

The Medstar Georgetown University Hospital Internal Review (IRB) approved this single institution retrospective study (IRB 09-510). Patients eligible for study inclusion had localized prostate cancer treated with SBRT. All patients provided informed consent prior to treatment. Baseline urinary symptoms (QOL) was assessed using the International Prostate Symptom Score (IPSS) with higher values (range 0-35) indicating more severe LUTS (18). An IPSS of 0–7 is mild, 8–19 is moderate and 20–35 is severe LUTS. Patients were eligible if they had localized prostate cancer treated with SBRT and completed the EPIC-26 Questionnaire. For this analysis, patients who did not complete the EPIC-26 at 1-month post-SBRT were excluded. No additional exclusions were made based on prior surgery or comorbidities.

### SBRT treatment planning and delivery

SBRT was delivered utilizing the CyberKnife robotic radiosurgical system (Accuray, Inc., Sunnyvale, CA, USA). Fiducial placement, treatment planning magnetic resonance imaging (MRI), and computed tomography (CT) simulation procedures have been previously described (2, 6). The clinical target volume (CTV) was defined as the prostate and proximal seminal vesicles to the point where the proximal seminal vesicles separate. The CTV was expanded 5 mm in all directions except 3 mm posteriorly to generate the planning target volume (PTV). Patients were treated to a prescription dose of 35-36.25 Gy to the PTV delivered in five fractions over two weeks based on treatment planning. The prescription isodose line was limited to ≥ 75% to restrict the maximum prostatic urethra dose to 133% of the prescription dose. Critical structure dose constraints were previously detailed by Chen et al.² For structures relevant to genitourinary toxicity, the maximum allowable dose was 37 Gy to both the bladder and the membranous urethra. Urinary symptoms were managed with alpha-antagonists and/or short steroid tapers. Patients were treated with tamsulosin 0.4 mg until moderate urinary symptoms abated. The standard protocol for a steroid taper was Decadron 4 mg in the morning for a week followed by 2 mg in the morning for a second week.

### Follow-up and statistical analysis

Patients completed the Expanded Prostate Cancer Index Composite (EPIC)-26 on the day of the first SBRT fraction and during routine follow-up one month and three months after the completion of SBRT (19). The EPIC-26 is a validated tool that measures associated bother with treatment related symptoms. Bothersome urinary frequency was assessed before and after treatment based on the patient reported response to Question 4e on the EPIC 26 (“How big a problem, if any, has the need to urinate frequently during the day been for you the last 4 weeks?”). Patients were seen at 1 month, 3 months, then at 6 months intervals for a year, then yearly for the rest of their lives.

To statistically compare changes between time points, the levels of responses were assigned a score, and the significance of the mean changes in the scores was assessed by paired t test. Wilcoxon Signed-Rank Test and chi-square analysis were used to assess differences in health-related quality of life (HRQOL) scores in comparison to baseline. Paired t-test was used to assess significance of the change in scores. The minimally important difference (MID) to assess for clinically significant change in HRQOL from baseline was set as half a standard deviation (SD). All analyses were performed using R statistical software.

Covariates were selected if they were statistically significant in univariate analysis and/or clinically relevant. For example, baseline AUA severity was included given its strong association with urinary frequency (p < 0.001) and clinical importance. Risk group was also included, since it was statistically significant in the univariate analysis (p = 0.04 for intermediate vs. low). Other variables (age, race, prostate volume, alpha antagonist use) were excluded as they were not significant in the univariate analysis and lacked strong clinical justification in this setting.

## Results

Baseline demographic and clinical patient characteristics are presented in **Table 1**. 1676 patients with localized prostate cancer were treated with prostate SBRT and followed for 3 months to evaluate acute toxicities. For this study, we limited the analysis to the 1078 patients who completed the EPIC-26 questionnaire at 1-month post-SBRT. The median patient age was 70 years old (IQR: 66-75). 58% self-identified as white and 35% as black. The median prostate volume was 39 cc, obtained through transrectal ultrasound (IQR: 28 –53). Using the D’Amico risk classification, 21% of patients were low risk, 66% of patients were intermediate risk, and 13% patients were high risk. The majority of patients were treated with 36.25 Gy in 7.25 Gy fractions. Baseline IPSS scores show that most patients had mild to moderate lower urinary tract symptoms with a high rate of alpha antagonist usage (72%) prior to receiving SBRT.

**Table 1:** Demographic and clinical characteristics of patients who did 1 month of follow-up.

Characteristics	No (%)			P value^b^
	All (n = 1078)	Urinary frequency^a^		
	No problem (n = 781)	Problem (n = 297)		
Age (y), Median (IQR)	70 (66-75)	70 (65-75)	71 (66-76)	0.4
<60	94 (9)	67 (9)	27 (9)	0.96
60-69	418 (39)	304 (39)	114 (38)	
70-79	463 (43)	337 (43)	126 (42)	
>80	103 (10)	73 (9)	30 (10)	
Race				0.2
White	625 (58)	463 (59)	162 (55)	
Black	380 (35)	270 (35)	110 (37)	
Other	73 (7)	48 (6)	25 (8)	
Risk Group				0.06
Low	229 (21)	155 (20)	74 (25)	
Intermediate	714 (66)	533 (68)	181 (61)	
High	135 (13)	93 (12)	42 (14)	
Alpha Antagonist Usage at Baseline				0.30^c^
Yes	778/1074 (72)	559/778 (72)	219/296 (74)	
No	281/1074 (26)	210/778 (27)	71/296 (24)	
Steroid	15/1074 (1)	9/778 (1)	6/296 (2)	
IPSS Severity at Baseline				**<0.01**
Mild (0-7)	362/1075 (34)	314/779 (40)	48/296 (2)	
Moderate (8-19)	572/1075 (53)	410/779 (55)	162/296 (47)	
Severe (20-35)	141/1075 (13)	55/779 (5)	86/296 (51)	
Prostate Volume (cc), Median (IQR)	39 (28-53)	39 (29-53)	38 (28-54)	0.7
	(n = 1070)	(n =775)	(n = 295)	

a Urinary frequency for each patient was determined by question 4E of the EPIC-26 questionnaire at 1 month of follow-up. “No problem” in the table includes “no,” “very small,” and “small” problems. “Problem” includes “moderate” and “big” problems.

b P values for the comparison between patients with or without urinary frequency problems using the t-test, Wilcoxon rank sum test, and chi-square test for normally, non-normally distributed continuous, and categorical variables, respectively.

c P-values based on Fisher’s exact test due to some small cell counts.

Bolded values are statistically significant using a p-value of 0.05.

**Figure 1:**
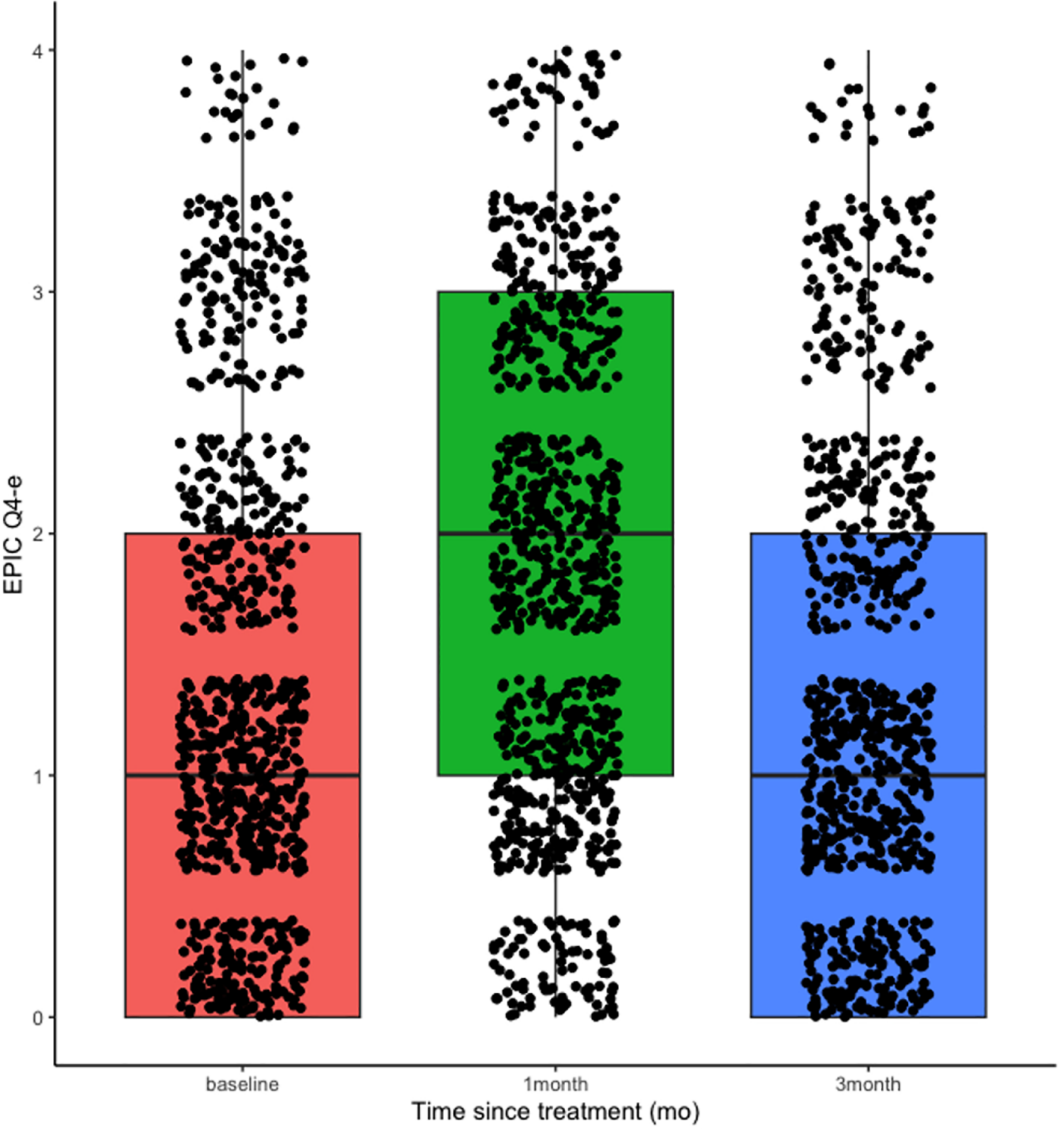
Mean scores for urinary frequency, assessed by Question 4e on the EPIC-26 (“How big a problem, if any, has the need to urinate frequently during the day been for you the last 4 weeks?”). Scores range from 0 (no problem) to 4 (big problem).

When looking at urinary frequency with question 4 on EPIC-26, patients endorsed increased urinary frequency at 1-month post-SBRT (mean of 2 on 4-point scale), with similar rates of frequency at 3 months compared to baseline (mean of 1 on 4-point scale; **Figure 1**). Patients with moderate and severe urinary symptoms per the baseline IPSS were more likely to endorse bothersome urinary frequency at one month post SBRT (EPIC-26 Q4E; OR 2.58 and 10.2 respectively) compared to those with mild symptoms (**Tables 2**, **3**). No significant differences were found in urinary frequency between patients who used and did not use an alpha antagonist at baseline (**Table 2**).

**Table 2:** Univariate logistic regression model predicting the risk of reporting urinary frequency in EPIC Q4-E.

Variable	β ± s.e.	Odds ratio	95% CI	P value
Age (y)				
Age (Ref: <60)	0.01 ± 0.01	1.01	0.99-1.03	0.3
60-69	-0.07 ± 0.25	0.93	0.57-1.55	0.8
70-79	-0.07 ± 0.25	0.93	0.57-1.54	0.8
>80	0.02 ± 0.31	1.02	0.55-1.90	0.95
Race (Ref: White)				
Black	0.15 ± 0.14	1.16	0.87-1.55	0.3
Other	0.4 ± 0.26	1.49	0.88-2.47	0.1
Risk Group (Ref: Low)				
Intermediate	-0.34 ± 0.17	0.71	0.52-0.99	**0.04**
High	0.06 ± 0.23	0.95	0.60-1.49	0.81
Alpha Antagonist Usage at Baseline (Ref: Yes)				
No	-0.15 ± 0.16	0.86	0.63-1.17	0.4
Steroid	0.53 ± 0.53	1.7	0.56-4.77	0.3
IPSS Severity at Baseline (Ref: Mild)				
Moderate (8-19)	0.95 ± 0.18	2.58	1.83-3.71	**<0.001**
Severe (20-35)	2.32 ± 0.23	10.2	6.54-16.2	**<0.001**
Prostate Volume (cc)	0.002 ± 0.002	1	1.00-1.01	0.6

Bolded values are statistically significant using a p-value of 0.05.

**Table 3:** Multivariate logistic regression model predicting the risk of reporting urinary frequency in EPIC Q4-E.

Variable	Univariate		Multivariate	
	Odds ratio (95% CI)	P value	Odds ratio (95% CI)	P value
Risk Group (Ref: Low)				
Intermediate	0.71 (0.52-0.99)	**0.04**	0.65 (0.46-0.92)	**0.02**
High	0.95 (0.60-1.49)	0.81	0.72 (0.44-1.17)	0.19
IPSS Severity at Baseline (Ref: Mild)				
Moderate	2.58 (1.83-3.71)	**<0.001**	2.66 (1.88-3.84)	**<0.001**
Severe	10.2 (6.54-16.2)	**<0.001**	10.5 (6.67-16.71)	**<0.001**

Risk Group vs. IPSS Severity at Baseline.

In our cohort, patients in the intermediate-risk group had a high association with having mild IPSS Severity score compared to Severe IPSS Severity score at baseline.

Bolded values are statistically significant using a p-value of 0.05.

## Discussion

Alpha-adrenergic antagonists have been routinely used therapeutically as well as prophylactically for radiation-related urinary symptoms and, more recently, were shown to be well-tolerated in patients undergoing SBRT (6–8). Alpha-adrenergic antagonists are designed to address the “voiding” category of LUTS by inducing smooth muscle relaxation in the prostate and bladder neck, thus reducing urinary obstruction (11). The “storage” category of LUTS may be better addressed with beta 3-adrenergic receptor agonists, medications designed to treat overactive bladder without as many anticholinergic side effects as antimuscarinic medications (12, 14). While our study does not specifically measure the efficacy of beta 3-adrenergic receptor agonists in men undergoing SBRT for prostate cancer, it provides a rationale for the future study of these medications in this patient population.

Mirabegron, a type of beta 3-adrenergic receptor agonist, has been shown to be effective in treating male patients with overactive bladder with minimal effects on urodynamics (16, 20). Adding mirabegron to an alpha-adrenergic antagonist improved frequent urination compared to an alpha-adrenergic antagonist alone (21). However, elevated blood pressure is common in elderly prostate cancer patients and blood pressure elevation is a known side effect of mirabegron (22).

Another beta 3-adrenergic receptor agonist, vibegron, was found to be effective and safe as an add-on therapy in men already receiving an alpha-adrenergic antagonist presenting with persistent storage symptoms (2). There has not yet been a study that examines the safety and utility of beta 3-adrenergic receptor agonists in treating LUTS in patients undergoing SBRT for prostate cancer. Importantly, vibegron does not increase blood pressure or interact with alpha-adrenergic antagonists, but it will be important to show low rates of acute urinary retention (2).

A prior study utilizing the EPIC-26 in the context of patients undergoing SBRT for prostate cancer found a 20% increase in the number of men who endorsed bothersome urinary frequency 1 week after the completion of treatment compared to pretreatment (3). This study highlights the prevalence of urinary frequency in men undergoing stereotactic body radiation therapy (SBRT) for clinically localized prostate cancer. Our findings indicate that SBRT treatment primarily exacerbates urinary urgency and frequency within the first month post-treatment, with a significant improvement by three months. This temporal pattern suggests that the acute effects of SBRT on the bladder may be distinct from more chronic prostate cancer treatments and may be particularly amenable to targeted interventions.

The analysis of IPSS symptom scores revealed that patients with moderate and severe urinary symptoms at baseline were more likely to report significant urinary urgency (IPSS Q4; ORs 2.69 and 5.72, respectively) and frequency (EPIC-CP Q4E; ORs 2.58 and 10.2, respectively) post-treatment. The relationship between baseline symptoms and post-treatment urgency and frequency reinforces that pre-existing voiding or storage dysfunction may serve as a risk factor for increased symptom burden after SBRT. The higher risk of urgency in those with moderate-to-severe baseline symptoms suggests that these patients may require closer monitoring and more aggressive symptom management strategies, including potential pharmacologic interventions.

While alpha-adrenergic antagonists such as tamsulosin have been widely used in SBRT patients to alleviate obstructive voiding symptoms (6, 7), our study did not find any protective benefit of baseline alpha-adrenergic antagonist usage in preventing urinary frequency. This is consistent with the literature suggesting that alpha-adrenergic antagonists are more effective in managing obstructive voiding dysfunction (e.g., weak stream, incomplete emptying), rather than storage symptoms, such as urgency and frequency (4–8). The lack of a protective effect from alpha antagonists further supports the hypothesis that medications targeting the beta-3 adrenergic receptor, which modulate bladder storage function, may be more appropriate for addressing storage-related LUTS in SBRT patients.

Beta-3 adrenergic receptor agonists, such as vibegron, have been shown to be safe and effective in improving symptoms of overactive bladder (OAB), including urinary urgency, in men (10, 11). Given that SBRT-related urinary urgency and frequency appears to peak at one month post-treatment, our findings suggest that the early addition of a beta-3 agonist in men at high risk for storage LUTS could provide a beneficial prophylactic strategy. This approach could be particularly valuable for patients with moderate to severe pre-treatment LUTS, as they appear to be more vulnerable to post-SBRT storage symptoms. Additionally, the lack of a significant difference in urinary frequency and urgency between patients who used and did not use an alpha antagonist suggests that addressing storage symptoms may require a different pharmacologic approach than the current standard of care in SBRT patients.

Our study has several strengths, including its large sample size (n=1078) and the use of validated questionnaires (IPSS and EPIC-26) to assess patient-reported outcomes. However, there are also several limitations to consider. First, the retrospective design of the study limits our ability to establish causal relationships between SBRT and the onset of urinary urgency. Additionally, because our cohort did not include patients who received other forms of radiation therapy or surgical interventions, the generalizability of our findings may be restricted to SBRT-treated individuals. Further, we assessed symptom severity using patient-reported outcomes rather than data obtained by urodynamic studies, which may provide a more comprehensive and objective understanding of the mechanisms underlying SBRT-induced LUTS.

## Conclusion

Our study demonstrates that urinary frequency is a common and significant side effect of stereotactic body radiation therapy (SBRT) in men with localized prostate cancer, particularly during the first month following treatment. Urinary symptoms appear to be most pronounced in patients with moderate to severe urinary symptoms at baseline, highlighting the need for tailored management strategies. The lack of a protective effect from alpha-adrenergic antagonists necessitates further research into whether alternative therapeutic strategies, such as beta-3 adrenergic agonists, may offer a more effective approach for managing storage-related LUTS in this patient population. Future prospective studies are needed to confirm these findings and evaluate the clinical benefit of beta-3 agonists in preventing or alleviating SBRT-induced urinary urgency and frequency.

## Data Availability

The raw data supporting the conclusions of this article will be made available by the authors, without undue reservation.
